# Effect of the different debonding strength of metal and ceramic brackets on the degree of enamel microcrack healing

**DOI:** 10.1590/2177-6709.26.3.e2119177.oar

**Published:** 2021-06-25

**Authors:** Piyaphong NIMPLOD, Ratchawan TANSALARAK, Tanapon SORNSUWAN

**Affiliations:** 1 Naresuan University, Department of Preventive Dentistry, Section of Orthodontics (Phitsanulok, Thailand).; 2 Naresuan University, Department of Restorative Dentistry, Section of Prosthodontics (Phitsanulok, Thailand).

**Keywords:** Debonding strength, Microcrack healing, Ceramic brackets, Indentation

## Abstract

**Objective:**

This study aims to determine shear debonding strength of metal and ceramic brackets, and the degree of enamel crack healing.

**Material and Methods:**

Extracted human maxillary premolars were flattened on the buccal surface, and randomly separated into five groups (n = 15). In control groups (groups 1 and 2), metal and ceramic brackets were bonded on flat polished enamel, while in experimental groups (groups 3 and 4), metal and ceramic brackets were bonded on the surface with boundary where corner cracks were created. Additionally, fifteen specimens (group 5) were also prepared for an indentation procedure with no bracket installation. The degree of crack healing was measured. All brackets were detached with a universal testing machine, and the adhesive remnant index (ARI) was also identified. Healing degree and apparent fracture toughness were then calculated.

**Results:**

Between groups with similar bracket types, there was no statistically significant difference in debonding strength. Regarding bracket types, ceramic brackets provided significantly higher debonding strength than metal brackets. There was a significant difference in ARI scores between metal and ceramic brackets. The corner cracks showed signs of healing in both horizontal and vertical directions. No statistically significant difference in the healing rates among the groups was found and the apparent fracture toughness increased from the initial to the final measurement.

**Conclusions:**

Within the limitations of this study, even though ceramic brackets required significantly higher debonding force compared to metal brackets, debonding stress was limited to the bonding site and did not affect the surrounding cracks on enamel.

## RESUMO

**Objetivo:**

O presente estudo teve como objetivo determinar a resistência ao cisalhamento de braquetes metálicos e cerâmicos, e o grau de reparo de fraturas no esmalte.

**Métodos:**

Pré-molares superiores, extraídos de humanos, foram aplainados na face vestibular e aleatoriamente divididos em cinco grupos (n = 15). Nos grupos controle (Grupos 1 e 2), os braquetes metálicos e cerâmicos foram colados em esmalte liso e polido; enquanto nos grupos experimentais (Grupos 3 e 4), os braquetes metálicos e cerâmicos foram colados em superfície delimitada, em cujos cantos foram criadas fissuras. Adicionalmente, foram também preparados 15 espécimes (Grupo 5) para um teste com indentação, sem a instalação de braquetes. O grau de reparo das fraturas foi avaliado. Todos os braquetes foram descolados usando uma máquina universal de testes, e o índice de adesivo remanescente (ARI) também foi avaliado. O grau de reparo e a tenacidade à fratura aparente foram então calculados.

**Resultados:**

Entre os grupos com o mesmo tipo de braquetes, não houve diferença estatisticamente significativa na força de descolagem. Com relação aos tipos de braquetes, os cerâmicos apresentaram uma força de descolagem significativamente maior do que os metálicos. Houve uma diferença significativa nos escores ARI entre os braquetes metálicos e os cerâmicos. As fraturas de canto mostraram sinais de reparo nos sentidos horizontal e vertical. Não foi detectada diferença estatisticamente significativa no grau de reparo entre os grupos, e a tenacidade à fratura aparente aumentou da mensuração inicial para a final.

**Conclusão:**

Considerando-se as limitações desse estudo, apesar de os braquetes cerâmicos necessitarem de força de descolagem significativamente maior do que os braquetes metálicos, a tensão de descolagem foi limitada ao sítio de colagem, não afetando as fraturas de esmalte ao redor.

## INTRODUCTION

Enamel cracks may be a consequence of several factors, including abnormalities in the maturation process, occlusal overloading, temperature variations, therapeutic procedures, and surface injuries from bracket removal - especially with the use of ceramic brackets.^1^ Several studies have determined that bonding of ceramic brackets to enamel provided higher bond strength when compared to conventional metal brackets.^2^ ^-^ ^4^ Such firm adhesion may cause some degree of micro surface damage in the form of crazing, crack or fracture on the enamel surface when brackets are removed.^5^

The enamel micro-defects after bracket removal are of great interest for orthodontists who use fixed orthodontic appliances.^6^ ^,^ ^7^ Presence of cracks may cause stain and plaque accumulation on the enamel and increase the risk for dental caries. Additionally, propagation of cracks may lead to more surface disintegration and structural loss.^8^ However, there is some evidence of enamel microcrack healing as a natural defense to prevent crack propagation to the dentin and to dental pulp.^9^

Few studies have evaluated enamel defects before bonding,^1,7^ and analyzed the presence of alteration of the control enamel microcracks before and after bonding brackets. Regarding the bracket types, there is a lack of knowledge on the relative microcrack characterization on debonded enamel after brackets removal. From the fractographical and mechanical aspects, the objectives of this study were to compare debonding strength and degree of crack repair on the debonded enamel after removal of metal and ceramic brackets.

## MATERIAL AND METHODS

Seventy five extracted human maxillary premolars were used for this research. These premolars were extracted due to orthodontic indications. These specimens, originated from both genders, between 16 and 40 years of age, were collected from patients at the surgical department in the School of Dentistry, Naresuan University, and private dental clinics, following an ethical approval protocol by the Institutional Review Board of Naresuan University. All premolars were caries-free, with no existing restorations nor root canal fillings, and with no sign of prominent cracks, abrasion or erosion. After extraction, all specimens were washed in running water to remove all blood and adhered tissue, stored in 0.1% thymol solution and then tested within a month of extraction, to reduce the potential for organic and inorganic losses.

After root separation using a high-speed carborundum disc, the specimens were positioned in a 2-cm diameter plastic ring with the most convex buccal surface of the tooth 2-3 mm above the surface of a self-cured acrylic resin, and then kept in 25°C water for 24 hours, for complete resin polymerization. A series of abrasive papers, with grits P1000, P1200, and 3-μm and 1-μm diamond pastes were consecutively used to standardize the curvature of the buccal surface of the teeth. The polishing protocol consisted of the use of a grinder polisher, driven with a 20-Newton force for 20 seconds, to achieve a flat area to bond the bracket base (9.28 ? 0.08 mm^2^ for metal and 10.38 ? 0.08 mm^2^ for ceramic brackets). The polishing was carried out horizontally relative to the cutting plane of the plastic ring.

All samples were randomly divided into five groups depending on the bracket type and with or without indentations:

» Group 1: Metal brackets bonded on non-indented specimens (n*=*15).
» Group 2: Ceramic brackets bonded on non-indented specimens (n*=*15).
» Group 3: Metal brackets bonded on indented specimens (n*=*15).
» Group 4: Ceramic brackets bonded on indented specimens (n*=*15).
» Group 5: Indented specimens with no brackets (n *=* 15).

**Figure 1:**
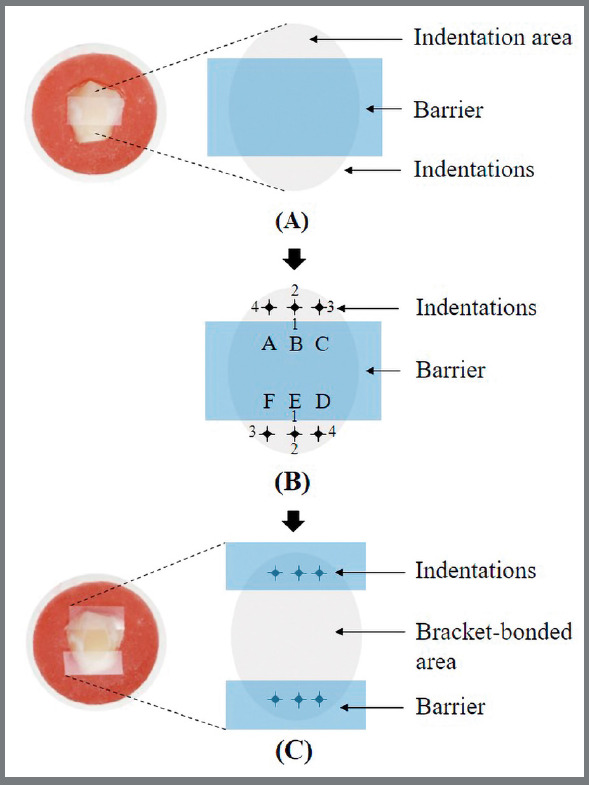
Schematic drawing of how to create a control bonded surface and the orientation of the indentations and corner cracks around the bracket base area. **A**) The first barrier used for separation before indentation, to locate indentation zone at the bracket boundary. **B**) Indentation making (nomenclature of the indented microcracks performed at the boundary according to their directions: A_1_, B_1_, C_1_, D_1_, E_1_, and F_1_ (centripetal vertical cracks); A_2_, B_2_, C_2_, D_2_, E_2_, and F_2_ (centrifugal vertical cracks); A_3_, B_3_, C_3_, D_3_, E_3_, and F_3_ (clockwise horizontal cracks); A_4_, B_4_, C_4_, D_4_, E_4_, and F_4_ (counterclockwise horizontal cracks). C) The second barrier used for protection of the indentations from resin infiltration before bracket attachment.

Before indention making in groups 3, 4, and 5, a four-millimeter-width rectangular barrier was attached to the middle of the polished area to separate the indented from the bonded areas (Fig 1A). Six micro-indentations were performed close to the edges of the barrier using a microhardness tester with a Vickers diamond indenter (Zwick/Roell; Indentec) loaded with a 500-gram force for 10 seconds (Fig 1B). Three indentations were created at the upper boundary including A, B, and C points from left to right, and another three indentations were created at the lower boundary including D, E, and F points from right to left (Fig 1B).

Each indentation created four corner cracks extending from the indentation. They were classified according to the direction of the crack in relation to the center of the bonded area, as follows; 1) Centripetal vertical crack, 2) Centrifugal vertical crack, 3) Clockwise horizontal crack, and 4) Counterclockwise horizontal crack (Fig 1B). The lengths of those twenty-four diagonal microcracks were measured with Vickers indentation diagonals at baseline using a binocular stereo microscope of the microhardness tester at magnifications of 100X and 400X before bracket bonding and also after debonding. By using machine software (Zwick/Roell) to draw measuring lines, which were calibrated with the size and depth of the indentation diagonal, between the indentation’s corner and the prominent crack tips, the crack length can be precisely measured.

**Figure 2:**
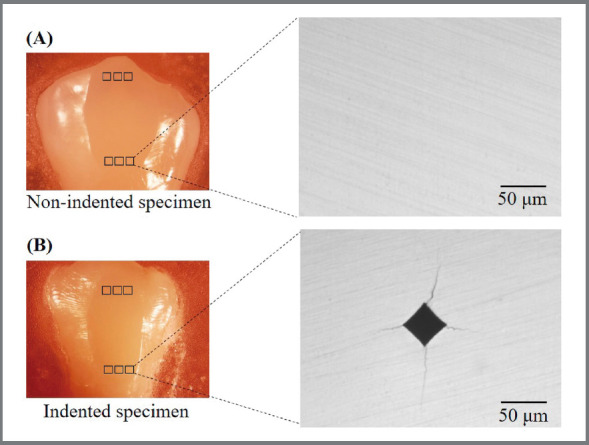
The samples with and without indentation: (A) Sample without indentations on polished enamel, (B) Sample with indentations on polished enamel.

**Figure 3:**
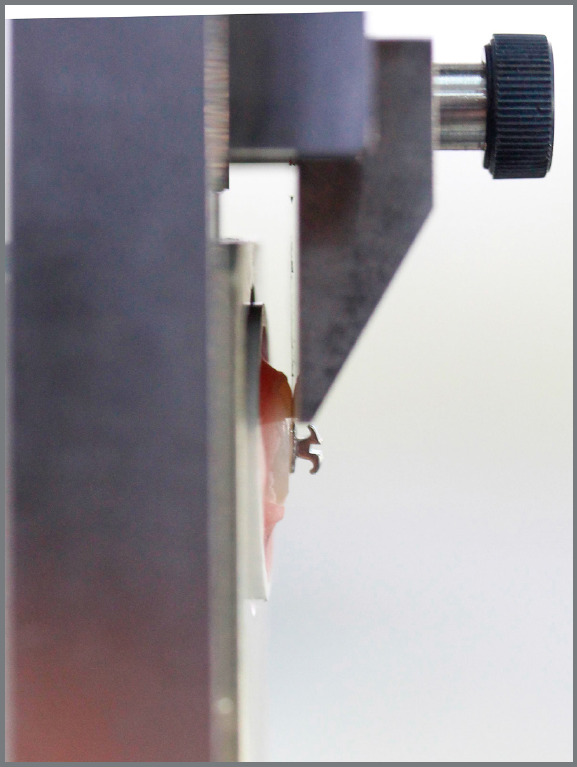
The orientation of a specimen in the universal testing machine.

The illustration of non-indented and indented specimens before bracket attachment is shown in Figure 2. The specimen’s unbonded areas were then covered with a barrier tape to avoid adhesive contamination on the microcracks and to control the bonding area (Fig 1C).The bonded surface of each specimen was prepared by etching with 37% phosphoric acid (3M Unitek) for 30 seconds, followed by 15-second water rinsing and 10-second drying with oil-free compressed air. The etched enamel was then painted with Transbond XT primer (3M Unitek) before application of Transbond XT paste (3M Unitek) to the bracket base. Metal (Gemini, 3M Unitek) and ceramic (Clarity, 3M Unitek) brackets of mandibular incisors were used. The brackets were placed and firmly pressed at the center of the polished surfaces. The excess adhesive was removed from the bracket base and light-activated for 3 seconds on each side of the metal bracket and 3 seconds through the ceramic bracket, according to the manufacturer’s instructions. After storage of all specimens in water for 24 hours (for complete resin polymerization), each bracket was debonded with a universal testing machine (Instron) with a crosshead speed of 1.0 mm/min, perpendicular to the bracket-enamel interface (Fig 3). The residual adhesive on the enamel surface and bracket base was assessed under a stereomicroscope, according to the modified adhesive remnant index (ARI).^10^

After the final measurement of corner cracks, healing rates in both vertical (*R*_*v*_ ) and horizontal (*R*_*h*_ ) directions were calculated according to the following equations:

$$R_{v}=\frac{V_{2}-V_{1}}{t}$$ (1)

$$R_{h}=\frac{H_{2}-H_{1}}{t}$$ (2)

where *V*_*1*_ *, V*_*2*_ *, H*_*1*_ *, H*_*2*_ , and *t* are the initial vertical crack length, final vertical crack length, initial horizontal crack length, final horizontal crack length, and healing time, respectively.

In addition, the crack length was used to analytically calculate the apparent fracture toughness (*K*_*c(app)*_ ) for each indentation according to the following equation:^11^

$$K_{c(app)}=0.0084{\left(\frac{E}{HV}\right)}^{2/5}\left(\frac{2F}{L}\right)\frac{1}{c^{1/2}}$$ (3)

where *HV, F, L*, and *c* are the Vickers hardness, indentation load, average diagonal length, and crack length, respectively. The elastic modulus (*E*) for enamel was obtained elsewhere.^12^

Standard descriptive statistics means and standard deviations were calculated for all parameters. The difference in the debonding strength and degree of crack alteration was compared statistically using a Kruskal-Wallis test. One-way analysis of variance (ANOVA) was used to determine the difference of healing rate of the microcracks and the apparent fracture toughness between groups. Any differences were further investigated using the *post-hoc* test. A statistically significant level was predetermined at 0.05 for all tests.

## RESULTS

A comparison of the debonding strengths within groups of similar bracket type and between metal and ceramic bracket groups is presented in Table1. The median debonding strengths of metal and ceramic groups were 23.06 MPa and 37.37 MPa for the non-indented groups, and 20.30 MPa and 31.85 MPa for the indented groups, respectively. There was no statistically significant difference in the strength between non-indented and indented specimens within the similar bracket type. However, ceramic brackets had significantly higher debonding strength than metal brackets (*p*< 0.001).

**Table 1:** Comparison of average debonding strength between groups after bracket debonding (µm).

Group	Median	Minimum	Maximum	Mean rank
1	23.06	11.73	25.05	19.13^a^
2	37.37	21.77	48.08	46.23^b^
3	20.30	13.32	26.60	14.13^a^
4	31.85	21.61	44.90	42.50^b^

Group 1:Bonding metal bracket on non-indented surface; Group 2: Bonding ceramic bracket on non-indented surface; Group 3: Bonding metal bracket on indented surface; Group 4: Bonding ceramic bracket on indented surface. Similar superscript letter on the column indicates no statistically significant difference.

Alteration of surface indented microcracks between metal and ceramic bracket groups is presented in Table 2. There was some degree of crack healing after removal of both metal and ceramic brackets, which are comparable to that for indentation on the surfaces without brackets. However, there were no statistically significant differences in the healing degree among the groups in both vertical (*p*=0.852) and horizontal (*p*=0.071) directions.

**Table 2:** Comparison of average microcrack alteration both in the vertical and horizontal directions between groups after bracket debonding (µm).

Crack directions	Bracket types	Median	Minimum	Maximum	Mean rank
Vertical	Metal	-11.5	-23.6	3.6	21.4^a^
	Ceramic	-8.1	-19.9	5.1	23.8^a^
	No bracket	-9.0	-17.2	-4.7	23.7^a^
Horizontal	Metal	-7.9	-14.5	-2.3	26.9^a^
	Ceramic	-10.5	-16.3	2.9	16.7^a^
	No bracket	-8.4	-13.7	-4.2	25.4^a^

Similar superscript letter on the column indicates no statistically significant difference.

Table 3 summarizes ARI scores of the debonded interfaces of all specimens. There were 13 specimens (43.3%) of metal brackets that failed at the enamel-adhesive interface (score 5), and twelve samples (40%) left adhesive on enamel surface more than 10% but less than 90% (score 3). However, there was no adhesive remnant on the enamel surface of the ceramic bracket group (score 5). Additionally, four samples bonded with the ceramic brackets (13.3%) presented enamel chipping.

**Table 3:** Frequency distribution of the adhesive remnant index (ARI scores).

Bracket types	ARI scores				Total	
	1	2	3	4	5	
Metal	1	0	12	4	13	30
Ceramic	0	0	0	0	30	30

Adhesive remnant index (ARI scores): 1 = all of the adhesive left on the enamel surface, 2 = more than 90% of the adhesive left on the enamel surface, 3 = more than 10% but less than 90% of the adhesive left on the enamel surface, 4 = less than 10% of adhesive left on the enamel surface, 5 = no adhesive left on the enamel surface.

Comparison of average microcrack healing rates among the groups after bracket debonding is presented in Table 4. No statistically significant difference was found among the groups (*p*= 0.792 for vertical and *p* = 0.215 for horizontal directions).

**Table 4:** Comparison of average microcrack healing rates between groups after bracket debonding (nm/s).

Bracket types	n	Direction of microcrack	
		Vertical	Horizontal
Metal	15	-0.10 ± 0.07	-0.08 ± 0.04
Ceramic	15	-0.10 ± 0.07	-0.11 ± 0.05
No bracket	15	-0.11 ± 0.04	-0.09 ± 0.03
p-value		0.792	0.215

Table 5 exhibits apparent fracture toughness at the initial and the final measurement between groups. Percentage of apparent fracture toughness increased from the initial to the final measurement as follows: 15.17, 14.57, and 11.36 in metal, ceramic, and no bracket groups, respectively.

**Table 5:** The average of apparent fracture toughness at the initial and final measurement (MPa.m^1/2^).

Bracket types	n	K_c(app)_		
		Initial K_c(app)_	Final K_c(app)_	% increase K_c(app)_
Metal	15	0.87 ± 0.07	1.00 ± 0.11	15.17 %
Ceramic	15	0.85 ± 0.06	0.98 ± 0.09	14.57 %
No bracket	15	0.79 ± 0.07	0.88 ± 0.12	11.36 %

## DISCUSSION

Since enamel cracks are difficult to detect clinically, control surface cracks were carried out before bracket attachment, to compare the effect of debonding shear stress on the cracks with both metal and ceramic brackets. However, there still were variations in the length and form of the control microcracks, i.e., crack branching, crack bridging or crack bifurcation in this study, even though the same loading protocol was used. This variation may be a consequence of the complex enamel prism orientations, as well as a range of mechanical behaviors that differ from the dentin-enamel junction to the enamel surface.^9^ ^,^ ^13^

Regarding shear debonding strength, there was no significant difference between the groups with the same bracket type, suggesting that the presence of surface microcracks on the enamel seems to not affect the bond strength between the bracket’s base and the surface enamel. However, for ceramic brackets, the value was significantly higher than for metal brackets and is comparatively higher than the value range of previous studies (from 10.4 ??#8197;4.1MPa to 21.67 ? 5.19 MPa^14^ ^-^ ^17^)and also the clinically recommended values to resist accidental bracket dislodgement (6 - 8 MPa^18^). The explanation for such high shear strength in this study may be the fact that flattened enamel may expose more enamel rods and thus, improve the bond quality.^19,20^ Even though standardization of the surface curvature might not be of clinical relevance, such a procedure was considered necessary to generate controlled straight corner cracks and to reduce uncertainty in the surface topography during the microcrack length measurements.

Interestingly, enamel fracture was observed in 13.3 percent of debonded ceramic brackets (4 out of 30 samples). It is noteworthy that enamel chipping occurred in the samples with an extremely high debonding strength (more than 40 MPa), which exceeded the cohesive strength of enamel.^21^ The high shear bond strength observed in this study may be due to the enamel surface preparation. Thus, this *in-vitro* study may represent an extreme situation of the debonding stress that might affect surrounding microcracks. This finding point out that polishing enamel before ceramic bracket installation, or repositioning, should be avoided, which is consistent with the relatively high incidence of enamel fractures that occurred after removing ceramic brackets, but none for metal brackets.^2-4^ Such high shear strength of ceramic brackets could lead to enamel surface loss, especially in a tooth with some existing enamel subsurface microcracks, or one previously weakened due to a fatigue response from an extensive restoration, or a tooth with root canal treatment. It has been reported that specimens with a high shear debond strength, of over 30 MPa, are likely to have surface enamel damage.^4^ Further research should focus on an optimal removing method for ceramic brackets that can reduce debonding force and protect the surface integrity of enamel.

**Figure 4:**
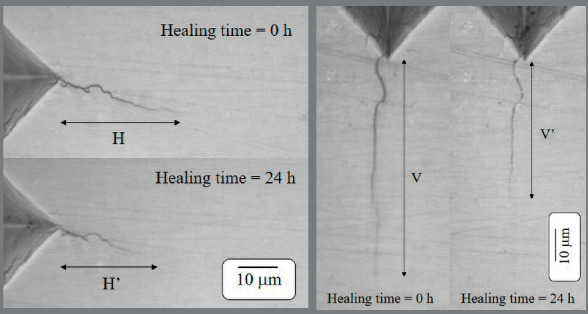
Illustrations of microcrack healing after debonding (H = horizontal crack length immediately after indentation, H’ = horizontal crack length 24 hours after bonding, V = vertical crack length immediately after indentation, V’ = vertical crack length 24 hours after bonding).

**Figure 5:**
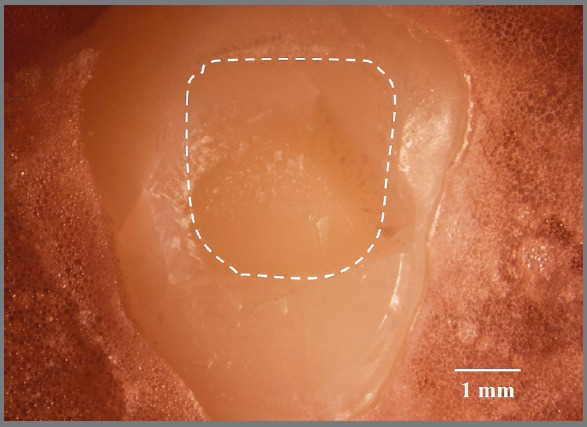
Illustration of enamel chipping after debonding a ceramic bracket (dotted line outlines the bracket bonded area).

The cracks partially repaired soon after the removal of the brackets (Fig 4). Interestingly, even with the highest debonding strength observed in the ceramic groups, the same healing rates of the corner cracks as those without brackets could still be found at the bracket’s boundary. The stress seems to be limited only to the bonded interface. This finding is consistent with the enamel chipping located on the proximity of incisal or gingival borders of the brackets in the ceramic group (Fig 5), and it is also consistent with a report in which finite element analysis of shear stress distribution in the enamel-adhesive interface was used. The researchers reported a pattern that was quite heterogeneous, and the stress concentration was limited to the upper and lower margins of the brackets.^22^

After indentation with a Vickers microhardness tester on the enamel surface, the length of the diagonal microcracks reduced with time. It has been reported that indented microcracks repaired around 9% of their initial length in the first 24 hours and reached a plateau level (10 % of the initial length) in 48 hours.^9^ The repair process may be the consequence of a viscoelastic recovery and extrinsic toughening mechanism of organic protein in enamel.^9^ When indentation on the enamel surface and microcrack generated, the crack does not penetrate the dentin but runs perpendicularly to the interface. The stress intensity initially increases with crack extension and the extrinsic toughening behavior from complex microstructure such as a range of enamel fracture toughness, crack bridging, and deflection act primarily behind the crack tip to reduce the crack-driving force.^23^ Soon after the stress intensity reaching the plateaus, the removal of the load and repairing process began. Enamel also exhibits significant viscoelasticity that can dissipate energy during deformation and fracture. During the fracture propagation, the organic protein was in the stretched stage, and created closure stress. Moreover, the importance of enamel protein on both crack resistance and repair was established in a study which found that deproteinization of enamel reduced fracture toughness from 1.23 ??#8197;0.20?#8197;MPa m^0.5^ to 0.95?#8197;??#8197;0.20?#8197;MPa?#8197;m^0.5^ (a 25% reduction).^13^

Even though enamel microcracks healing is a normal process on a vital tooth preventing crack propagation to the dentin and dental pulp, this healing process can be found in extracted tooth. According to ISO/TS 11405:2015, the teeth that have been extracted for longer than six months may undergo degenerative changes in enamel and dentinal protein.^24^ However, the teeth used in this study have been tested within a month after extraction. Therefore, the remaining organic protein in enamel still had an influence on crack closure stress, and healing process occurred.

**Figure 6:**
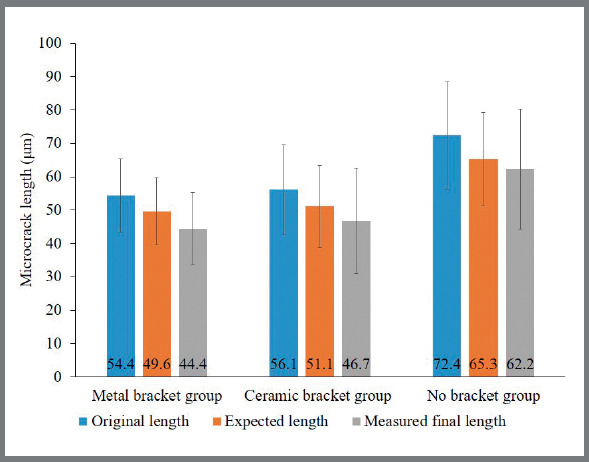
Comparison of 24-hour vertical crack lengths: original length (before bracket removal), final length (after bracket removal) and expected length calculated with original length subtracted from a reported degree of healing.

**Figure 7:**
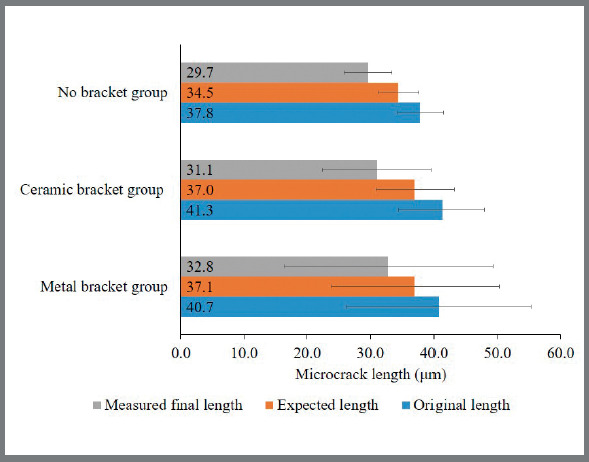
Comparison of 24-hour horizontal crack lengths: original length (before bracket removal), final length (after bracket removal) and expected length calculated with original length subtracted from a reported degree of healing.

Since in this study the corner cracks were remeasured at least 24 hours after indentation, the crack length was a resultant of crack healing combined with the stress at the bracket’s boundary. The expected length was then calculated by using a healing degree of 9%, as suggested in the literature.^9^ The original and expected 24-hour crack lengths, both in the vertical and the horizontal directions are presented in Figures 6 and 7, respectively. It was observed in this study that the cracks healed slightly more than expected in both directions. Even for the specimens with high debonding strength, such as those with enamel chipping, the stress on the surrounding area during removal of a bracket was minimal and did not extend the microcracks. Additionally, the healing degree in this study might have been more efficient because the outer enamel was polished, since the fracture toughness, as well as the organic content, was found to increase from the surface enamel to the dentin-enamel junction.^9^ ^,^ ^25^

Comparing the differences between the crack lengths alone might be insufficient to determine the effect of shearing stress on the surrounding microcrack behavior, because of the time lag between each measurement. The average time elapsed between the crack measurement resulting from indentation and the remeasurement after debonding was 26.19 ± 3.69 hours. Although ceramic brackets had higher debonding strengths compared to metal brackets, there was no significant difference in the degree of healing between the groups. These similar healing rates confirm that bracket removing stress did not affect the healing process of boundary enamel microcracks.

The stress intensity factor (*KI*) is another mechanical parameter used to describe resistance of any material to critical crack growth. The critical value of *KI* or fracture toughness (*Kc*) of enamel can be evaluated by an indentation approach.^25^ It has been reported that 10% reduction of the apparent fracture toughness is associated with the degree of microcrack healing in the enamel surface. That reduction in *K*_*c(app)*_ is consistent with bridging by the organic matrix in enamel that can be defined as follows:^9^

$$K_{b(\text{protein})}=2\sigma_{b}f_{b}{\left(\frac{2l_{b}}{\pi }\right)}^{1/2}$$ (4)

Where $\sigma_{b}$ is the nominal bridging stress on the protein matrix (assumed to be equivalent to the yield strength of protein, *f*_*b*_ is the area fraction of protein matrix bridging ligaments, and *l*_*b*_ is the bridging zone length. For this study, due to the crack reduction, there was an increase in *K*_*c(app)*_ from the initial indentation to the final observation, of approximately 15% (Table 4). This higher reduction is probably due to the investigated area on the enamel surface, which was located in the inner part of the buccal enamel, where a greater content of organic matter exists. This layer of enamel may have higher organic bridging stress and bridging zone length, as expressed in equation 3. Consequently, the degree of crack healing in this study is larger than that found by Rivera et al.^9^

For the ARI scores, combination groups of the same bracket type (groups 1 and 3, as well as groups 2 and 4) were performed due to no statistically significant difference of debonding force between groups within similar bracket type. Bond failure for brackets was found to be more prevalent at the enamel-adhesive interface, especially in ceramic brackets (100%) (Table 1). The result might be due to a higher bond strength between the ceramic bracket bases and the adhesive. The predominant failure type of debonded ceramic brackets was found to be at the bracket-adhesive interface.^15,17^ This kind of failure is beneficial to the enamel surface because it is left intact, although more time is required to remove the adhesive remnant.^26^ On the contrary, there is a higher probability of enamel damage if the unit fails at the enamel-adhesive interface.^27^

All brackets used in this study were subjected to the shear strength test with a universal testing machine to deliver shear force. The unilateral axial load applied to the bonding surface by this testing machine creates pure shear stress, which might differ from removing pliers used clinically.^17^ Consequently, the stress generated by a bracket removing plier is not directly comparable to the condition used in this study.^28^ Debonding strength exerted by bracket removing plier has been reported to be 30% less than the shear strength delivered by the universal testing machine.^29^

There are some limitations to this study. Firstly, a standardized laboratory setup may be extrapolated to a complex clinical situation, e.g., changes in temperature, humidity, acidity, mechanical and masticatory stress on brackets. Besides, moisture control *in vitro* is superior to *in vivo*. Secondly, the delayed measurement of microcrack length after bracket debonding could not be a real-time crack analysis. Thirdly, the location of the crack tip was difficult to identify by using the microscope, which resulted in an approximate error of 30-70 μm. Lastly, the limited number of crack formations and observation of crack initiation was only located in the surrounding area, which might not represent all the stress conditions within the bonded interface. Finite element analysis, or a real-time crack propagation study, might be used to determine the stress concentration at the crack tip, as well as a microtomography study of the bonded interface immediately after bracket removal.

## CONCLUSIONS

Within the limitations of this study, the conclusions are as follows:

Removal of ceramic brackets required a higher debonding strength and was more susceptible to enamel fracture than with metal brackets. 

The surrounding cracks partially healed after bracket debonding.

The debonding stress from bracket removal was quite localized and did not affect the healing degree of surrounding microcracks.
